# Supplementary material for: Validating the bifactor structure of the Ruminative Thought Style Questionnaire—A psychometric study
Source: PLoS One. 2021 Jul 26;16(7):e0254986. doi: 10.1371/journal.pone.0254986 (PMC8312922; doi:10.1371/journal.pone.0254986)
Supplement: S1 Table — Total Sample: N = 1123; Males: N = 505 (45%); Females: N = 618 (55%). RTSQ, Ruminative Thought Style Questionnaire total score; CES-D, The Center for Epidemiologic Studies Depression Scale; BSI_GSI, Brief Symptom Inventory General Symptom Index; PfT, Problem-focused thoughts factor of the Ruminative Thought Style Questionnaire; CT, Counterfactual thinking factor of the Ruminative Thought Style Questionnaire; RT, Repetitive thoughts factor of the Ruminative Thought Style Questionnaire; AT, Anticipatory thoughts factor of the Ruminative Thought Style Questionnaire. *p < .05.; **p < .01.; ***p < .001. (DOCX) [file pone.0254986.s001.docx]

**Supporting information.**

**Validating the Bifactor Structure of the Ruminative Thought Style Questionnaire - a Psychometric Study**

S1 Table. Standardized regression weights between the RTSQ total scores and covariates in Study 1.

|  | total sample | | | | | male | | | | | female | | | | |
| --- | --- | --- | --- | --- | --- | --- | --- | --- | --- | --- | --- | --- | --- | --- | --- |
| Covariates | RTSQ | PfT | CT | RT | AT | RTSQ | PfT | CT | RT | AT | RTSQ | PfT | CT | RT | AT |
| Gender | 0.08^*^ | 0.09^*^ | -0.04 | 0.23^***^ | 0.26^***^ | - | - | - | - | - | - | - | - | - | - |
| Age | -0.01 | -0.01 | -0.09^*^ | 0.05 | -0.04 | 0.04 | -0.07 | -0.01 | 0.06 | -0.05 | -0.04 | 0.06 | -0.11^*^ | 0.02 | -0.04 |
| CES-D | 0.39^***^ | 0.32^***^ | 0.16^**^ | 0.17^***^ | 0.02 | 0.38^***^ | 0.35^***^ | 0.16^*^ | 0.15 | 0.06 | 0.38^***^ | 0.30^***^ | 0.16^**^ | 0.20^***^ | -0.02 |
| R^2^ | 0.16^***^ | 0.11^***^ | 0.03^***^ | 0.09^***^ | 0.07^***^ | 0.15^***^ | 0.12^***^ | 0.03^***^ | 0.03^***^ | 0.01^***^ | 0.15^***^ | 0.09^***^ | 0.04^***^ | 0.04^***^ | 0.002^***^ |

Total Sample: N =1123; Males: N =505 (45%); Females: N =618 (55%). RTSQ, Ruminative Thought Style Questionnaire total score; CES-D, The Center for Epidemiologic Studies Depression Scale; BSI_GSI, Brief Symptom Inventory General Symptom Index; PfT, Problem-focused thoughts factor of the Ruminative Thought Style Questionnaire; CT, Counterfactual thinking factor of the Ruminative Thought Style Questionnaire; RT, Repetitive thoughts factor of the Ruminative Thought Style Questionnaire; AT, Anticipatory thoughts factor of the Ruminative Thought Style Questionnaire. ^*^p<.05.; ^**^p<.01.; ^***^p<.001.
