# Supplementary material for: Validating the bifactor structure of the Ruminative Thought Style Questionnaire—A psychometric study
Source: PLoS One. 2021 Jul 26;16(7):e0254986. doi: 10.1371/journal.pone.0254986 (PMC8312922; doi:10.1371/journal.pone.0254986)
Supplement: S2 Table — Model 1 = One factor CFA; Model 2 = Second-order four factor CFA; Model 3 = bifactor CFA; Model 4 = bifactor ESEM; AIC, Akaike Information Criteria; χ2, chi-square test statistic; df, degree of freedom; CFI, Comparative Fit Index; TLI, Tucker-Lewis Index; RMSEA, Root Mean Squared Error of Approximation; CI, confidence interval; SRMR, Standardized Root Mean Square Residual. (DOCX) [file pone.0254986.s002.docx]

**Supporting information.**

**Validating the Bifactor Structure of the Ruminative Thought Style Questionnaire - a Psychometric Study**

**S2 Table. Factor Analyses of four measurement models of the Ruminative Thought Style Questionnaire in Study 2.**

|  | AIC/BIC | χ^2^ | df | CFI | TLI | RMSEA | 90% CI | SRMR |
| --- | --- | --- | --- | --- | --- | --- | --- | --- |
| Model 1 | 22927.011/  23153.110 | 886.192 | 170 | .689 | .653 | .115 | .11-.12 | .084 |
| Model 2 | 16665.819/  16850.467 | 225.810 | 86 | .917 | .899 | .071 | .06-08 | .061 |
| Model 3 | 21198.533/  21466.084 | 365.921 | 138 | .899 | .874 | .072 | .06-.08 | .057 |
| Model 4 | 21074.542/  21538.045 | 169.632 | 86 | .963 | .926 | .055 | .04-.07 | .025 |

Model 1= One factor CFA; Model 2= Second-order four factor CFA; Model 3= bifactor CFA; Model 4= bifactor ESEM; AIC, Akaike Information Criteria; χ2, chi-square test statistic; df, degree of freedom; CFI, Comparative Fit Index; TLI, Tucker-Lewis Index; RMSEA, Root Mean Squared Error of Approximation; CI, confidence interval; SRMR, Standardized Root Mean Square Residual.
