# Supplementary material for: Validating the bifactor structure of the Ruminative Thought Style Questionnaire—A psychometric study
Source: PLoS One. 2021 Jul 26;16(7):e0254986. doi: 10.1371/journal.pone.0254986 (PMC8312922; doi:10.1371/journal.pone.0254986)
Supplement: S3 Table — RT, Repetitive thoughts factor of the Ruminative Thought Style Questionnaire; CT, Counterfactual thinking factor of the Ruminative Thought Style Questionnaire; PfT, Problem-focused thoughts factor of the Ruminative Thought Style Questionnaire; AT, Anticipatory thoughts factor of the Ruminative Thought Style Questionnaire. (DOCX) [file pone.0254986.s003.docx]

**Supporting information.**

**Validating the Bifactor Structure of the Ruminative Thought Style Questionnaire - a Psychometric Study**

**S3 Table. Alpha and Omega reliability for the bifactor ESEM (Model 4) in Study 2.**

| Model 4 | Omega total (ω) | Omega hierarchical (ω_h_) | Cronbach α |
| --- | --- | --- | --- |
| General bifactor | .935 | .846 | .902 |
| RT | .856 | .397 | .841 |
| CT | .814 | .448 | .800 |
| PfT | .817 | .331 | .804 |
| AT | .763 | .429 | .745 |

RT, Repetitive thoughts factor of the Ruminative Thought Style Questionnaire; CT, Counterfactual thinking factor of the Ruminative Thought Style Questionnaire; PfT, Problem-focused thoughts factor of the Ruminative Thought Style Questionnaire; AT, Anticipatory thoughts factor of the Ruminative Thought Style Questionnaire.
