# Supplementary material for: Validating the bifactor structure of the Ruminative Thought Style Questionnaire—A psychometric study
Source: PLoS One. 2021 Jul 26;16(7):e0254986. doi: 10.1371/journal.pone.0254986 (PMC8312922; doi:10.1371/journal.pone.0254986)
Supplement: Attachment — Submitted filename: Answer letter to reviewers RTSQ_uploaded.docx [file pone.0254986.s004.docx]

Dear Dr. Frantisek Sudzina,

We thank you that you are willing to consider a revision. We would like to take the opportunity and resubmit the revised version of our manuscript, entitled *“Validating the Bifactor Structure of the Ruminative Thought Style Questionnaire - a Psychometric Study”*.

***Please, find below our detailed answers to the reviewers’ comments written in blue***. In the revised manuscript the original text is presented in black, ***modifications are indicated by tracked changes*** ***mode in MS Word.*** We hope that we have managed to address each of the reviewers’ concerns.

Sincerely,
the authors

**Review Comments to the Author**

**Reviewer #1: The present study attempts to investigate the factor structure of the RTSQ using ESEM bifactor in two large samples, as well as gender invariance and criterion-related validity. There are a number of technical/methodological problems with this article. Additionally, the overall scientific contribution is not clear. I would suggest making the paper a brief report.**

Thank you for taking the time to review our manuscript! When completing the review, we put great emphasis on making the introduction more concise and the scientific contribution clearer. However, we find that the psychometric details of our paper are crucial for understanding our work, thus - although we tried our best to make it as short and parsimonious as possible - we believe it is more understandable in the form of a research article than a brief report.

**To begin, the introduction/setup of the paper was far too long. The authors should remember that factor analysis does not ‘uncover reality’. In this vein, detailed descriptions of who found which factor structure are likely not needed.**

The rationale for presenting the factor structure and reliability of RTSQ in this detailed manner was that the structure of the RTSQ seems to slightly differ in various cultural and linguistic settings. Although we are aware that these kinds of analysis do not uncover reality, we wanted to point out that this heterogeneity could impact the results. At the same time, we admit that the introduction part was too long, thus we substantially shortened the introduction.

**In text:**

When examining the factor structure of the RTSQ, the authors found the single-factor solution the most adequate. The retained 20 items showed high internal consistency (Cronbach’s α= .92 and Cronbach’s α= .87).

(...)

They removed five items (items 10,15,16,18,19) and suggested a second-order four-factor solution, with subscales named as Problem-focused thoughts (Items 9,11-14), Counterfactual thinking (Items 5-8), Repetitive thoughts (Items 1-4) and Anticipatory thoughts (Items 17, 20) where the items loaded on the four subscales together formulated a general higher-order rumination factor Thus, the authors concluded that the RTSQ measures rumination as a rather multidimensional, multifaceted construct.

(...)

Among the factor extraction methods described above, only the bifactor solution is capable of separating how much of the item response variance derives from a single latent variable, and how much is attributable to its subgroups, which is a crucial aspect when improving a scale that may contribute to better interpret the trait itself.

(...)

**Moreover, what is not clear is the ultimate contribution of this study. What is the clinical relevance of finding that the RTSQ has this structure or that? How does one versus the other finding in terms of structure impact what you do in the clinic?**

Thank you for outlining this! The goal of our study was to find a useful and reliable instrument that covers different facets of the multidimensional rumination construct, beyond its connection with depression. We also added further thoughts to clarify the ultimate goal of examining the factor structure of the RTSQ to the General Discussion.

In text (page 34, lines 551-561):

As rumination is a transdiagnostic risk factor to psychopathology (73) that should be a target of interventions (74), it is crucial to define reliable rumination measures for assessment and treatment purposes - in case of the RTSQ, to disentangle whether the global score or the subscales are more advised to use for such purposes. This is especially important in case of a university student sample, as that age range is considered a sensitive period for the emergence of psychological problems such as mood disorders (75), for which rumination is considered a substantial risk factor, primarily among women (6), highlighting the relevance of examining gender invariance of rumination measures such as the RTSQ. Moreover, rumination has been the target of specific therapeutic interventions (76), hence a reliable rumination scale could help to accurately measure post-therapeutic change in ruminative tendencies.

**Notably, page 12, line 179 – predicting depression from the RTSQ factors is criterion-related validity, not convergent validity (that would be another measure of rumination).**

Thank you for this remark. We carefully revised these definitions and agree that convergent validity rather reflects the relationship between the RTSQ and another rumination measure. However, criterion validity is the ability of a measure to predict real-life outcomes or symptoms, thus are more relevant for diagnostic scales (Strauss & Smith, 2009), where the criterion is best assessed through physiological or behavioral measures, rather than another self-report scale representing latent constructs (McDonald, 2005). Construct validity, however, is a broader category comprising many facets of validity (Messick, 1995). McDonald (2005) defines construct validity as follows: “Construct validity refers to how well a measure is associated with measures of other latent concepts that are theorized to have [causal relationships](https://www.sciencedirect.com/topics/computer-science/causal-relationship), or constructs, with one another.” In our understanding the association between rumination and depression falls into this category, as they appear to be causally related (e.g. (Nolen-Hoeksema & Girgus, 1994). Thus, when referring to the relationship between the RTSQ and a depression scale, we corrected the word “convergent validity” to “construct validity” in the manuscript (page 1, lines 6-7; page 12, line 201; page 17, line 310; page 25, line 379; page 25, line 390; page 29, line 458).

**Methods:**

**Please document – at least briefly – the validation procedure for Hungarian measures in terms of translation/back-translation and cross-cultural construct validity previously found, if any**.

Thank you for outlining this issue, we included the description of the validation procedure of the Hungarian RTSQ in the manuscript. The total score of the Hungarian RTSQ demonstrated high internal consistency in two independent studies (Cronbach α = .88; Cronbach α =.91) (Kocsel et al., 2019; Kovács et al., 2021).

In text (page 13, lines 213-218):

Two independent researchers translated the RTSQ from English to Hungarian. Differences were resolved by discussion and consensus with the help of a third native Hungarian-speaking researcher who used to live in an English-speaking country for years. Then a fourth researcher backtranslated the Hungarian version to English. A native English-speaking psychologist reviewed the two versions and found that the backtranslation adequately reflected the meaning of the original items.

Cross-cultural results about the factor structure of the RTSQ can be found in Table 1 of the Introduction. We completed their description in the text for more clarity.

In text (page 10, lines 144-146):

Although results about the cross-cultural validity of the RTSQ are scarce, Bravo et al. and Walsh et al. found that the RTSQ demonstrated measurement invariance across U.S, Spanish and Argentinian samples (32), and across Chinese and Australian samples (29).

Investigating the cross-cultural properties of the Hungarian RTSQ was beyond the scope of this work – we outlined this as a limitation in the General discussion.

Page 38, lines 633-637:

Another limitation is that the models we wished to replicate were tested on different translations of the RTSQ (e.g. English and Serbian), whereas we tested the factor structure of the Hungarian translation. Although beyond the scope of our work, it would be crucial for future studies to investigate whether diverging results reflect inconsistency in the measurement of rumination as a construct per se, or rather reflect idiomatic differences.

**It seems a little odd to use fit indices to arbitrate between models, especially when using a bifactor and ESEM. The bifactor is already a very general model and has a tendency to overfit – there’s quite a bit of discussion in the literature about it. ESEM compounds this problem further; in other words, there’s basically no way for the ESEM bifactor not to come out as the best-fitting model. The authors should at least discuss some of the auxiliary ways of arbitrating between models – content of general/specific factors, whether a factor is ‘collapsing in’ on itself, whether it’s driven by a single factor loading (see the AT factor), etc in this context. Next, the authors could have reasonably stopped at Model 3 – it’s more interpretable and fits fine (although I’d have to look at the specific factor loadings to be sure).**

Thank you for outlining these issues! We have expanded the explanation in the Discussion of Study 1 why Model 4 is preferred over the other models. We kept the same factor structure proposed by Mihic et al. (2019) in both Model 3 and Model 4, according to which the AT factor comprises three items. The only difference between Model 3 and Model 4 is that we allowed for cross-loadings in case of Model 4, as we believe that forcing items to load on one single factor is arbitrary when the subscales do not seem to be independent based on item content. Factor loadings improved significantly with this approach; we have included a table containing the specific factor loadings of Model 3 to review.

Standardized factor loadings of the bifactor CFA model of the RTSQ (Model 3).

| Items | Bifactor | RT | CT | PfT | AT |
| --- | --- | --- | --- | --- | --- |
| 1. I find that my mind goes over things again and again | .49 | .60 |  |  |  |
| 2. When I have a problem, it will gnaw on my mind for a long time | .57 | .58 |  |  |  |
| 3. I find that some thoughts come to my mind over and over throughout the day | .56 | .62 |  |  |  |
| 4. I can’t stop thinking about some things | .58 | .33 |  |  |  |
| 5. When I am expecting to meet someone, I will imagine every possible scenario and conversation | .58 |  |  |  | .25 |
| 6. I tend to replay past events as I would have liked them to happen | .51 |  | .52 |  |  |
| 7. I find myself daydreaming about things I wish I had done | .54 |  | .60 |  |  |
| 8. When I feel I have had a bad interaction with someone, I tend to imagine various scenarios where I would have acted differently | .61 |  | .45 |  |  |
| 9. When trying to solve a complicated problem, I find that I just keep coming back to the beginning without ever finding a solution | .55 |  |  | .26 |  |
| 10. If there is an important event coming up, I think about it so much that I work myself up | .63 |  |  |  | -.21 |
| 11. I have never been able to distract myself from unwanted thoughts | .61 |  |  | .34 |  |
| 12. Even if I think about a problem for hours, I still have a hard time coming to a clear understanding | .45 |  |  | .74 |  |
| 13. It is very difficult for me to come to a clear conclusion about some problems, no matter how much I think about it | .46 |  |  | .64 |  |
| 14. Sometimes I realise I have been sitting and thinking about something for hours | .50 |  |  | .23 |  |
| 15. When I am trying to work out a problem, it is like I have a long debate in my mind where I keep going over different points | .52 |  |  | .10 |  |
| 17. When I am looking forward to an exciting event, thoughts of it interfere with what I am working on | .65 |  |  |  | -.30 |
| 18. Sometimes even during a conversation, I find unrelated thoughts popping into my head. | .61 | -.08 |  |  |  |
| 19. When I have an important conversation coming up, I tend to go over it in my mind again and again | .71 |  |  |  | .25 |
| 20. If I have an important event coming up, I can’t stop thinking about it | .63 |  |  |  |  |

*Note. RTSQ= Ruminative Thought Style Questionnaire. RT= Repetitive thoughts factor of the Ruminative Thought Style Questionnaire. CT=Counterfactual thinking factor of the Ruminative Thought Style Questionnaire. PfT= Problem-focused thoughts factor of the Ruminative Thought Style Questionnaire. AT=Anticipatory thoughts factor of the Ruminative Thought Style Questionnaire.*

In text (page 27, lines 407-429):

Considering the guidelines of Hu & Bentler (61), Model 3 did not demonstrate an adequate fit due to their low CFI and TLI values, thus we could not accept it as our best model. However, when subscales do not represent distinct entities, forcing items to load on one single factor will not represent the construct accurately (37). RTSQ was aimed to measure rumination globally (3) – assuming that its subscales are not interrelated seems arbitrary and contradicts its theoretical background. ESEM allows for item cross-loadings, thus it is preferred in case of complex scales that lack consensus about their factor structure (35), such as the RTSQ. Additionally, although a bifactor model may not be appropriate for all measures, especially those with homogenous item content, it is considered the best model for those instruments where we *theoretically* expect a strong common trait behind the responses, but also a multidimensionality caused by well-defined clusters (54). Therefore, we proposed a fourth model, a bifactor ESEM solution containing 19 items on the general factor, and 14 items on the subscales that demonstrated the best model fit. This finding appears to reconcile the original unidimensional suggestion of the authors of the RTSQ (7) with the multifactorial proposition of Tanner et al. (24), in line with the findings of Mihić et al. (27). Allowing for cross-loadings substantially improved the factor scores of the model too, supporting that this approach represents the structure of the RTSQ better. Moreover, the exploratory feature of the ESEM may unravel where ambiguous items belong to. For instance, by replicating the model proposed by Mihic and colleagues, we only allowed item 10 to be loaded on the general factor, however, the ESEM approach revealed that it may belong to the AT subscale, that is in line with its content (“If there is an important event coming up, I think about it so much that I work myself up.”).

**Additionally, why use 90% rather than 95% CI?**

The 90% confidence interval refers to the RMSEA value. In case of RMSEA, it is more conventional to set the confidence level to 90% because it is a one-sided confidence interval, as opposed to the more typical 95% CIs, which are generally two-sided (Kline, 2015).

**Finally, the cross-loadings don’t seem to be reported (Table 3).**

Thank you for your remark, we added the cross-loadings of Model 4 to Table 3.

In text (page 19-20):

Table 3. Standardized factor loadings of the bifactor ESEM of the RTSQ.

| Items | Bifactor | RT | CT | PfT | AT |
| --- | --- | --- | --- | --- | --- |
| 1. I find that my mind goes over things again and again | **.50** | **.61** | -0.05 | -0.04 | -0.07 |
| 2. When I have a problem, it will gnaw on my mind for a long time | **.55** | **.61** | -0.01 | 0.07 | -0.03 |
| 3. I find that some thoughts come to my mind over and over throughout the day | **.56** | **.63** | -0.05 | -0.02 | -0.05 |
| 4. I can’t stop thinking about some things | **.52** | **.38** | 0.09 | 0.02 | 0.09 |
| 5. When I am expecting to meet someone, I will imagine every possible scenario and conversation | **.55** | 0.08 | 0.23 | -0.09 | 0.01 |
| 6. I tend to replay past events as I would have liked them to happen | **.49** | -0.05 | **.58** | 0.03 | -0.01 |
| 7. I find myself daydreaming about things I wish I had done | **.53** | 0.01 | **.57** | 0.05 | -0.07 |
| 8. When I feel I have had a bad interaction with someone, I tend to imagine various scenarios where I would have acted differently | **.60** | 0.004 | **.48** | -0.06 | -0.03 |
| 9. When trying to solve a complicated problem, I find that I just keep coming back to the beginning without ever finding a solution | **.56** | <0.01 | 0.16 | .**23** | -0.04 |
| 10. If there is an important event coming up, I think about it so much that I work myself up | **.53** | 0.07 | 0.01 | 0.10 | 0.43 |
| 11. I have never been able to distract myself from unwanted thoughts | **.56** | 0.14 | 0.03 | **.37** | 0.14 |
| 12. Even if I think about a problem for hours, I still have a hard time coming to a clear understanding | **.49** | -0.05 | 0.03 | **.71** | -0.03 |
| 13. It is very difficult for me to come to a clear conclusion about some problems, no matter how much I think about it | **.55** | -0.01 | -0.09 | **.57** | -0.10 |
| 14. Sometimes I realise I have been sitting and thinking about something for hours | **.64** | -0.06 | -0.06 | 0.11 | -0.17 |
| 15. When I am trying to work out a problem, it is like I have a long debate in my mind where I keep going over different points | **.72** | -0.06 | -0.22 | -0.09 | -0.18 |
| 17. When I am looking forward to an exciting event, thoughts of it interfere with what I am working on | **.58** | -0.09 | -0.03 | 0.07 | **.41** |
| 18. Sometimes even during a conversation, I find unrelated thoughts popping into my head. | **.56** | -0.04 | 0.04 | 0.02 | 0.19 |
| 19. When I have an important conversation coming up, I tend to go over it in my mind again and again | **.65** | 0.04 | 0.06 | -0.16 | **.28** |
| 20. If I have an important event coming up, I can’t stop thinking about it | **.55** | 0.01 | -0.07 | 0.03 | **.66** |

*Note. RTSQ= Ruminative Thought Style Questionnaire. RT= Repetitive thoughts factor of the Ruminative Thought Style Questionnaire. CT=Counterfactual thinking factor of the Ruminative Thought Style Questionnaire. PfT= Problem-focused thoughts factor of the Ruminative Thought Style Questionnaire. AT=Anticipatory thoughts factor of the Ruminative Thought Style Questionnaire.*

**Notably, please go through the measure carefully and clean out any criterion contamination with depression items or criteria. Some of the content still appears overlapping. Additionally, in Table 3, items 18 and 20 seem to be the same.**

Being biased by depressive symptoms was a major critique of the 22-item Ruminative Response Scale, especially its depression subscale, containing items that show substantial overlap with the Beck Depression Inventory, e.g.: “Think about how sad you feel”; “Think about your feelings of fatigue and achiness”; “Think about how you don’t seem to feel anything anymore”; “Think about how you don’t feel up to doing anything” (Treynor et al., 2003). After carefully reviewing the items of the RTSQ, we don’t seem to find any items that overlap with depression items or criteria. Thank you for drawing our attention to the typo in Table 3, we corrected item 18 (see in Table 3 below our previous answer).

**There appear to be issues with the gender invariance models. First, the authors don’t include the AIC across models. Second, it appears that the likelihood ratio tests across models are significant, which would indicate a worsening fit across more constrained models. As such, I don’t think the authors can argue for gender invariance.**

Thank you for your remark, we added the AIC, along with the BIC values of the invariance models to Table 5. Invariance was also supported by AIC and BIC showing decreases (or very low increases) up to the inclusion of invariance constraints on the latent variances and covariances. The information criteria were increased when invariance constraints were imposed on the items’ intercepts. The results are in line with the finding of Morin and colleagues (Morin et al., 2016), indicating that changes in goodness-of-fit indices tend to be untrustworthy indicators of latent variances-covariances and latent mean invariances.

In text (page 24):

Table 5. Testing measurement invariance of the RTSQ across genders

| Model | χ2(df) | AIC/BIC | RMSEA | RMSEA 90%CI | TLI | CFI | Model comparison | ∆RMSEA | ∆CFI |
| --- | --- | --- | --- | --- | --- | --- | --- | --- | --- |
| A.) Configural invariance | 430.787(172)* | 73913.023/  75148.868 | .052 | [.046-.058] | .932 | .966 | - | - | - |
| B.) Metric/weak invariance | 526.271(242)* | 73891.454/  74775.636 | .046 | [.040-.051] | .947 | .962 | B-A | -.006 | -.004 |
| C.) Scalar/strong invariance | 555.960(256)* | 73893.351/  74707.200 | .046 | [.040-.051] | .947 | .960 | C-B | <.001 | -.002 |
| D.) Strict invariance | 555.960(256)* | 73893.351/  74707.200 | .046 | [.040-.051] | .947 | .960 | D-C | <.001 | <.001 |
| E.) Var-covariance invariance | 618.019(290)* | 73898.995/  74542.036 | .045 | [.040-.050] | .949 | .957 | E-D | -.001 | -.003 |
| F.) Invariance of latent means | 724.556(295)* | 74008.382/  74626.304 | .051 | [.046-.056] | .934 | .943 | F-E | .006 | -.014 |

*Note. RTSQ = Ruminative Thought Style Questionnaire. χ² = chi-square test statistics; df= degree of freedom; RMSEA = Root Mean Squared Error of Approximation; CFI = Comparative Fit Index; TLI= Tucker-Lewis Index, CI= confidence interval.*

In text (page 16, lines 302-307):

The tested non-nested models were compared with Aikaike Information Criteria (AIC) and Bayesian Information Criteria (BIC). In the past, the model with the lowest AIC or BIC value were considered as the best fitting model to the data, but subsequent studies pointed out that information criteria should be considered as a rough guideline that should be used in combination with parameter estimates and theoretical adequacy, especially outside of the CFA framework, such as ESEM (56,57).

(...)

In text (page 28, lines 439-446):

Based on the chi-squared difference statistics, the invariance was only supported for the configural model and not for more constrained models. However, given that the chi-square difference test is often criticized because of its sensitivity to the sample size and to normal distribution (49), additional analyses of other indices is worthwhile (60). Cheung and Rensvold (63) recommended that CFI or RMSEA delta values be investigated before conclusions are made about the lack of invariance. Decreases of .01 or more in CFI across the models provide more certainty that the hypothesis should be rejected (58).

**Finally, why not examine predictive invariance (predicting depression) across gender? Why not examine whether the specific factors predict a criterion variable above and beyond the general factor within the model, rather than just using sum scores?**

Thank you for this suggestion, we included this analysis as supporting material.

In text (page 26, lines 401-404):

To further investigate the construct validity of RTSQ, we estimated a model with covariates to explore the total score and the subscales’ relationship with depression across gender. The standardized regression weights for the total sample and by gender can be found in Table S1 of the supporting material.

In text (Page 1 of Supporting material):

S1 Table. Standardized regression weights between RTSQ total scores and covariates in Study 1

|  | total sample | | | | | male | | | | | female | | | | |
| --- | --- | --- | --- | --- | --- | --- | --- | --- | --- | --- | --- | --- | --- | --- | --- |
| Covariates | RTSQ | PfT | CT | RT | AT | RTSQ | PfT | CT | RT | AT | RTSQ | PfT | CT | RT | AT |
| Gender | 0.08* | 0.09* | -0.04 | 0.23*** | 0.26*** | - | - | - | - | - | - | - | - | - | - |
| Age | -0.01 | -0.01 | -0.09* | 0.05 | -0.04 | 0.04 | -0.07 | -0.01 | 0.06 | -0.05 | -0.04 | 0.06 | -0.11* | 0.02 | -0.04 |
| CES-D | 0.39*** | 0.32*** | 0.16** | 0.17*** | 0.02 | 0.38*** | 0.35*** | 0.16* | 0.15 | 0.06 | 0.38*** | 0.30*** | 0.16** | 0.20*** | -0.02 |
| R^2^ | 0.16*** | 0.11*** | 0.03*** | 0.09*** | 0.07*** | 0.15*** | 0.12*** | 0.03*** | 0.03*** | 0.01*** | 0.15*** | 0.09*** | 0.04*** | 0.04*** | 0.002*** |

*Note. Total Sample: N =1123; Males: N =505 (45%); Females: N =618 (55%). RTSQ = Ruminative Thought Style Questionnaire total score; CES-D= The Center for Epidemiologic Studies Depression Scale; BSI_GSI= Brief Symptom Inventory General Symptom Index; PfT= Problem-focused thoughts factor of the Ruminative Thought Style Questionnaire; CT=Counterfactual thinking factor of the Ruminative Thought Style Questionnaire; RT= Repetitive thoughts factor of the Ruminative Thought Style Questionnaire; AT=Anticipatory thoughts factor of the Ruminative Thought Style Questionnaire. *p<.05.; **p<.01.; ***p<.001.*

(...)

In text (page 29, lines 459-463):

Furthermore, the regression model (included in the Supporting material) showed that depressive symptoms were significantly associated with the RTSQ total score and subscales, except for AT, which is plausible given the more positive and future-oriented content of its items. The strongest predictor of depressive symptoms was the RTSQ total score, indicating the relevance of the total score in clinical settings.

**Study 2: why did the authors not replicate the same models as in Study 1?**

We did replicate the models of Study 1 in Study 2 where Model 4 showed the best model fit. However, we only included their fit indices as supporting information to make our paper more concise. This information can be found here in our manuscript:

In text (page 33, lines 530-533):
Similarly to Study 1, the bifactor ESEM showed good fit to the data in Study 2 (χ^2^= 169.632, df=86, CFI=.96, TLI=.93, RMSEA=.06, SRMR=.03). Further details about CFA models and internal consistency can be found in S1 Table and S2 Table of supplementary material.

Here are the fit indices provided as supporting information:

In text (Page 2-3 of Supporting material):

**S2 Table. Factor Analyses of four measurement models of the Ruminative Thought Style Questionnaire in Study 2.**

|  | AIC/BIC | χ^2^ | df | CFI | TLI | RMSEA | 90% CI | SRMR |
| --- | --- | --- | --- | --- | --- | --- | --- | --- |
| Model 1 | 22927.011/  23153.110 | 886.192 | 170 | .689 | .653 | .115 | .11-.12 | .084 |
| Model 2 | 16665.819/  16850.467 | 225.810 | 86 | .917 | .899 | .071 | .06-08 | .061 |
| Model 3 | 21198.533/  21466.084 | 365.921 | 138 | .899 | .874 | .072 | .06-.08 | .057 |
| Model 4 | 21074.542/  21538.045 | 169.632 | 86 | .963 | .926 | .055 | .04-.07 | .025 |

*Note. Model 1= One factor CFA; Model 2=Second-order four factor CFA; Model 3= bifactor CFA; Model 4= bifactor ESEM; AIC=Akaike Information Criteria; χ^2^ = chi-square test statistic; df= degree of freedom; CFI= Comparative Fit Index; TLI= Tucker-Lewis Index; RMSEA= Root Mean Squared Error of Approximation; CI= confidence interval; SRMR= Standardized Root Mean Square Residual*

**Also, why was discriminant validity (e.g., some externalizing metric) not included?**

Thank you for outlining this idea, it would be very interesting to examine the discriminant validity of RTSQ. At the same time, rumination is considered a transdiagnostic risk factor to psychopathology, thus it has been associated with a wide array of psychological (and somatic) problems (e.g. (Ehring & Watkins, 2008; Watkins & Roberts, 2020b). As such, it is quite challenging to find a completely unrelated construct, therefore we considered this would be beyond the scope of our paper. For instance, even though externalizing symptoms may appear unrelated to rumination as it is typically associated with internalizing symptoms, empirical evidence supports that rumination weakly but significantly correlates with externalizing behavior (Clancy et al., 2016; du Pont et al., 2018; McLaughlin et al., 2014; Nolen-Hoeksema et al., 2007; Nolen-Hoeksema & Watkins, 2011). Nonetheless, we agree that discriminant validity of the RTSQ would be interesting to measure in future studies, e.g. with the BIG-5 factor Agreeableness that may be an unrelated construct (Kocsel et al., 2017). We noted this as a limitation in the General Discussion.

In text:

Furthermore, we did not examine the discriminant validity of the RTSQ. Although rumination is associated with a wide array of psychological (and somatic) problems (e.g. 15,71), Agreeableness, a Big Five personality trait defined as accommodating, amiable, friendly, and trustworthy (Shi et al., 2018) appears to be an unrelated construct (Kocsel et al., 2017) that could be used for such purposes. However, this was beyond the scope of our current paper.

**Age should have been controlled for, given the range.**

We did not control for age originally, as we did not consider the age range particularly wide (mean age=23.28, SD=2.93 years). Now we included a regression model in the manuscript where age is controlled for. Although it is a significant predictor of the RTSQ score, it does not substantially change the associations presented in our manuscript.

In text:

We also included a regression model in Table 9 to be able to control for gender and age, and to see whether ZSDS is significantly associated with RTSQ even after controlling for the RRS subscales.

Table 9. Association between RTSQ total scores, trait rumination (measured by RRS) and depressed mood (ZSDS scores) after controlling for gender and age.

|  | B | SE | β | R^2^ | ΔR^2^ |
| --- | --- | --- | --- | --- | --- |
| Step 1 |  |  |  | .397 |  |
| Constant | 50.700 | 9.469 |  |  |  |
| gender | .621 | 2.369 | .012 |  |  |
| Age | -.994 | .299 | -.153** |  |  |
| RRS Brooding | 3.369 | .281 | .559*** |  |  |
| RRS Reflection | .803 | .291 | .128** |  |  |
| Step 2 |  |  |  | .479 | .081*** |
| Constant | 23.115 | 9.710 |  |  |  |
| gender | .172 | 2.209 | .003 |  |  |
| age | -.717 | .282 | -.111* |  |  |
| RRS Brooding | 2.301 | .305 | .381*** |  |  |
| RRS Reflection | .797 | .272 | .127** |  |  |
| ZSDS total | .854 | .126 | .342*** |  |  |

*Note. RTSQ total= Ruminative Thought Style Questionnaire total score; RRS=Ruminative Response Scale, ZSDS= Zung Self-rating Depression Scale. *p<.05; ** p<.01; ***p<.001.*

We really appreciate your critics, comments, and questions regarding our work. They really helped us improve our manuscript. We also hope that we have managed to address your concerns.

**Reviewer #2: This manuscript investigates psychometric properties of the Ruminative Thought Style Questionnaire (RTSQ) among university students. Authors found that (1) the factor structure of the RTSQ was best described with a 19-item bifactor Exploratory Structural Equation Modelling (ESEM), and (2) the RTSQ was congruent with the RRS. This study appears to have a good methodology; however, it is unclear what the rumination would be.**

Thank you for your time reviewing our manuscript!

**Introduction**

**1.The most important problem was the lack of information about the definition of "Rumination." What is the gold standard for this word?**

We agree with your point - there is no consensus about the definition of rumination. We completed the Introduction of our manuscript with this information:

In text (page 2, lines 21-28):

A gold standard or consensus about the definition of rumination is lacking. As Smith and Alloy (1) in their review pointed out, there are many different conceptualizations of rumination. These theories differ in several dimensions: a) the degree to which they consider rumination as a stable construct or as a transitive, state-like phenomenon (2,3); b) whether it is the frequency or the actual content of ruminative thought that is more important (4); c) how rumination relates to other similar or partially overlapping constructs such as negative automatic thoughts, repetitive negative thinking or self-focused attention 3,5).

(...)

In text (pages 5-6, lines 112-118):

While Nolen-Hoeksema narrowly defined rumination as a potential response (or response style) to depressed mood, Tanner and colleagues (24) provided a more integrative definition, highlighting the multifaceted nature of rumination. Similarly to Brinker and Dozois (7), Tanner et al. also argue that repetitivity, intrusiveness or uncontrollability are core elements of rumination which might suggest non-productivity, but they also argued that that in some cases, rumination might be useful in identifying strategies and/or resources to cope with future eventualities (24).

**2. Although authors state that “Ruminative response, accompanied by other psychosocial factors, appears to aggravate depressed mood from early adolescence in case of women (ref.10)”, are there any reports that have shown clinical significance using the RTSQ (score) in university students? The significance of the RTSQ total score in the target population should be justified in the Introduction section, since the authors state that they want to see if the RTSQ total score is a valid measure of rumination**.

Thank you for outlining this. We included further support for the relevance of RTSQ among university samples.

In text (page 4, lines 87-91):

Moreover, the authors of the RTSQ conducted a daily diary study on an undergraduate sample where they found that the RTSQ prospectively predicted depressed mood, even after controlling for baseline depressive symptoms, highlighting the clinical significance of rumination among university students, that appears to be well captured by the RTSQ.

(...)

In text (page 5, lines 109-111):

In a recent empirical study Bravo et al. (25) found that the Problem-focused Thoughts subscale of the RTSQ mediated the relationship between depressive symptoms and drinking as a means of coping, supporting the scale’s relevance among university students.

**3.The authors raise the ambiguity of the concept of rumination, and issues of differences in structural validity between studies as a problem, but there is not enough information on a problem for content validity of the RTSQ.**

Thank you for this suggestion, we added notes on the content validity of the RTSQ in the Introduction.

In text (page 6, lines 118-126):

Regarding content validity, the authors of the RTSQ focused on conceptualizing rumination as a generic thought pattern, emphasizing its recurrent and intermittent feature rather than its negative valence and past-oriented tendency. Additionally, the four subscales identified by Tanner and colleagues may reflect those core aspects of rumination that the RTSQ can capture. Past psychometric studies (26,27) consistently found the Repetitive thought subscale (items 1-4), indicating that the RTSQ reflects the repetitive nature of ruminative thinking well. However, other important aspects of rumination, such as automaticity, involuntariness, and goal insensitivity (15) may be reflected less by the items of the RTSQ.

**Methods & Results;**

**4.Sample collection: How were the participants collected for this study？ At least, eligibility criteria need to be shown. (Study1 & Study2)**

Thank you for outlining this, we completed the information on eligibility criteria for Study 1 and Study 2.

In text - Study 1 (page 13, lines 223-229):

Students were recruited from several university dormitories, who participated in the study on a voluntary basis. Potential participants were contacted in person in their dormitories by research assistants in a systematic manner, where they could fill out the self‐report measures on paper in their room at their own pace after providing written informed consent. Inclusion criteria were age of 18 years or older and active student status at the university, no further restrictions applied.  In all institutions, refusal to participate in the whole study was approximately 5%.

In text - Study 2 (page 29, lines 472-473):

Eligibility criteria included being 18 years old or older with no previous history of mental or neurological illness.

**5.Convergent validity needs to be assessed in separate analyses for men and women, respectively. (Study1)**

Thank you for this suggestion, we added this information to Table 7.

In text (page 26):

Table 7. Correlations between RTSQ, BSI and CES-D scores (Study 1).

|  | total sample | | female | | male | |
| --- | --- | --- | --- | --- | --- | --- |
|  | BSI_GSI | CES-D | BSI_GSI | CES-D | BSI_GSI | CES-D |
| RTSQ total | .53 | .46 | .53 | .44 | .50 | .45 |
| PfT | .50 | .45 | .52 | .44 | .51 | .44 |
| CT | .36 | .33 | .39 | .34 | .30 | .31 |
| RT | .41 | .37 | .46 | .38 | .44 | .38 |
| AT | .35 | .29 | .36 | .27 | .35 | .31 |
| CES-D | .71 | 1.00 | .71 | 1.00 | .69 | 1.00 |

*Note. Total Sample: N =1123; Males: N =505 (45%); Females: N =618 (55%). RTSQ total= Ruminative Thought Style Questionnaire total score; RTSQ subscales: RT= Repetitive thoughts; CT=Counterfactual thinking; PfT= Problem-focused thoughts; AT=Anticipatory thoughts;* *BSI_GSI= Brief Symptom Inventory General Symptom Index; CES-D= The Center for Epidemiologic Studies Depression Scale; M=mean; SD=standard deviation. All correlations are significant at p < .001 level.*

**6.Why did the authors examine the correlation between the CES-D and RTSQ in Study 1, while the correlation between the ZSDS and RTSQ in Study 2? Not only the ZSDS but also the CES-D scale are widely used to evaluate depressive symptom severity, with higher scores reflecting increased symptom severity. Please elaborate on why the depression scale was changed between studies 1 and 2. (Study1 & Study2)**

Thank you for outlining this, we completed our manuscript with this explanation.

In text (pages 29-30, lines 477-483):

In Study 2 instead of the CES-D, we applied the Zung Self-Rating Depression Scale, another widely used and reliable measure of depression. Empirical findings support that the two scales are interchangeable (Ruiz-Grosso et al., 2012; Saltukoğlu & Tatar, 2017). Appling another measure of depression enabled us to see whether the findings of Study 1 regarding the associations of RTSQ and depression would be generalizable to another depression scale, reducing the probability that any association between the two constructs is due to item-level biases.

**7.Why did authors examine the relationship between the RTSQ scores and each subscale score, but not the total RRS** **score? (Study2)**

Thank you for your note, we added the information regarding the RRS total score to Table 8.

In text (page 31-32):

Table 8. Pearson correlations between measures along with the means, standard deviations and Cronbach’s alphas (Study 2).

|  | 1. | 2. | 3. | 4. | 5. | 6. | 7. | 8. | 9. |
| --- | --- | --- | --- | --- | --- | --- | --- | --- | --- |
| 1.RTSQ total(α=.90) |  |  |  |  |  |  |  |  |  |
| 2. PfT(α=.79) | .79** |  |  |  |  |  |  |  |  |
| 3. CT (α=.80) | .70** | .45** |  |  |  |  |  |  |  |
| 4. RT(α=.84) | .79** | .55** | .46** |  |  |  |  |  |  |
| 5. AT(α=.75) | .72** | .43** | .33** | .47** |  |  |  |  |  |
| 6. RRS total | .54** | .49** | .40** | .50** | .33** |  |  |  |  |
| 7.RRS Brooding(α=.71) | .60** | .50** | .52** | .50** | .32** | .78* |  |  |  |
| 8.RRS Reflective pondering(α=.71) | .23** | .14* | .10 | .26** | .14* | .78** | .21** |  |  |
| . ZSDS(α=.67) | .58** | .63** | .40** | .45** | .28** | .41** | .54** | .09 |  |
| Total sample, M (SD) | 75.41 (19.19) | 11.12 (4.87) | 11.47 (4.74) | 20.59 (4.93) | 12.90  (4.11) | 23.59 (4.93) | 10.73 (3.17) | 12.87 (3.16) | 39.28 (7.70) |
| Males, M (SD) | 72.88 (20.24) | 10.56 (4.89) | 11.79 (5.31) | 19.63 (5.02) | 12.06  (4.02) | 23.23 (5.33) | 10.21 (3.16) | 13.02 (3.48) | 37.78 (6.32) |
| Females, M (SD) | 75.90 (18.99) | 11.23 (4.88) | 11.41 (4.63) | 20.78 (4.90) | 13.06  (4.12) | 23.66 (4.85) | 10.83 (3.17) | 12.84 (3.10) | 39.57 (7.92) |
| t-statistics (p) | 1.03 (.31) | 0.90  (.37) | 0.53 (.60) | 1.54  (.13) | 1.61  (.11) | 0.58 (.56) | 1.29 (.20) | 0.38 (.70) | 1.51 (.13) |

*Note. Total Sample: N=320; Males: N =52 (16%); Females: N =268 (84%); RTSQ total= Ruminative Thought Style Questionnaire total score; RTSQ subscales: RT= Repetitive thoughts; CT=Counterfactual thinking; PfT= Problem-focused thoughts; AT=Anticipatory thoughts; RRS=Ruminative Response Scale, ZSDS= Zung Self-rating Depression Scale; M=mean; SD= standard deviation; *p<.05; ** p<.001.*

**Discussion;**

**8.In the method section, the word "Reliabilty" is used when explaining Cronbachα, but the word "Internal consistency" is used in the discussion section. The different ways of expressing the same meaning can be confusing. According to the COSMIN, the word "Reliability" can have several meaning, which may cause misinterpretation.**

Thank you for this suggestion, we changed the word “reliability” to “internal consistency” throughout the manuscript.

In text:

The test’s Hungarian adaptation demonstrated good internal consistency in a previous study (Cronbach α = .82) (Urbán et al., 2014), as well as in this sample (Cronbach α= .77).

(...)

**Internal consistency of the best fitting model (bifactor ESEM)**

The Cronbach αs of the total score of the RTSQ and its subscales demonstrated good internal consistency, in line with previous findings (Mihić et al., 2019; Tanner et al., 2013). In order to eliminate the errors in the estimation of internal consistency, the omega total and omega hierarchical coefficients were calculated (for details see Table 4.).

Table 4. Alpha and omega internal consistency for the bifactor ESEM of the RTSQ (Model 4).

(...)

Both subscales of the Hungarian adaptation demonstrated good internal consistency in a previous study (Cronbach αs: .71 and .73, respectively) (Kokonyei et al., 2016), as well as in the current sample (Cronbach αs were .71 for brooding and reflective pondering).

(...)

Further details about CFA models and internal consistency can be found in S2 Table and S3 Table of the supporting information.

Thank you very much for your critics, comments and questions regarding our work that helped us to improve our manuscript substantially. We also hope that we have managed to address your concerns.
